# Rehabilitation of Rare Neurological Complications of COVID-19 Infection in Health Resort Settings

**DOI:** 10.7759/cureus.58221

**Published:** 2024-04-14

**Authors:** Nadina Kurtanović, Ena Gogić, Edina Tanović, Damir Čelik, Elmina Mulić Hadžiavdić

**Affiliations:** 1 Department of Physical Medicine and Rehabilitation, Health Institution Spa Gata - Bihać, Bihać, BIH; 2 Clinic for Physical Medicine and Rehabilitation, Clinical Center University of Sarajevo, Sarajevo, BIH; 3 Clinic for Physical Medicine and Rehabilitation, University Clinical Center - Tuzla, Tuzla, BIH

**Keywords:** covid-19, rehabilitation outcomes, acute transverse myelitis, chronic inflammatory demyelinating polyradiculoneuropathy, guillain-barré syndrome, rehabilitation, neurological complications

## Abstract

There is increasing evidence of neurological involvement in patients with coronavirus disease. Reports of neurological manifestations include altered mental status, Guillain-Barré syndrome (GBS) and its forms, encephalopathy, psychosis, neurocognitive (dementia) syndrome, ischemic strokes, intracerebral hemorrhage, and acute transverse myelitis. We present three patients with rare neurological manifestations of the COVID-19 disease, with a special focus on rehabilitation in a health resort setting. Outcomes were evaluated based on neurological examination and the modified Barthel index. We highlight the importance of an interdisciplinary approach to reduce disability and improve functionality and quality of life.

## Introduction

Although the respiratory system is the primary site of involvement in most COVID-19 patients, there is increasing evidence indicating neurological complications in individuals with coronavirus disease. Neurological manifestations reported in the literature include altered mental status, Guillain-Barré syndrome (GBS) and its variants, encephalopathy, psychosis, neurocognitive (dementia-like) syndrome, ischemic strokes, intracerebral hemorrhage, and transverse myelitis [1]. A range of studies, both experimental and clinical, confirm that SARS-CoV-2 has neuroinvasive potential and can cause damage to the brain and peripheral nervous system. Microglia and neuroinflammation are considered factors of neurotropism [2]. These findings call for the development of strategies for managing and treating such cases.

Rehabilitation has proven to be successful in the acute phase of COVID-19 patients, in mild, moderate, and stable severe hospitalized patients [3]. In post-acute COVID-19 conditions, including neuroCOVID, rehabilitation is also recommended. The current data regarding rehabilitation outcomes of SARS-CoV-2-associated neurological complications are limited. We present three patients with rare neurological manifestations of SARS‑CoV‑2 infection regarding rehabilitation in health resort settings. Health resorts utilize a variety of interventions, including balneotherapy, hydrotherapy, and climatotherapy, alongside other rehabilitation strategies such as therapeutic massage, water massage, various forms of exercise, and other modalities of physical therapy [ 4]. For the evaluation of the outcomes, we used the modified Barthel index developed by Shah et al. [5]. The first case is acute motor axonal neuropathy, a form of Guillian-Barré syndrome. The second case is chronic demyelinating polyradiculoneuropathy, and the third case is acute transverse myelitis.

## Case presentation

Case one

A 57-year-old female, with a history of depression and previous lumbal spine stenosis operation, developed symptoms of mild infective syndrome, which manifested with pain in muscles, headache, and fatigue. She tested positive for COVID-19 via a polymerase chain reaction (PCR) test ordered by her family medicine doctor. Fifteen days after being infected, she experienced tingles in her legs, followed by weakness in her legs, and frequently fell while walking. After admission to the local hospital, the patient was referred to the neurological clinic for further diagnosis. She was diagnosed with acute motor axonal polyneuropathy (AMAN) and was treated with pulse corticosteroid therapy. Upon discharge from the neurological clinic, the patient underwent rehabilitation in our institution. On admission, she is in a wheelchair and needs help in transfers from wheelchair to bed. Gross motor strength scores on manual muscle testing were as follows: hip flexors and extensors 3/5, knee flexors and extensors 3/5, and dorsal and plantar flexors of feet 2/5. Superficial sensory functions (including pain, touch, and temperature) and deep sensory functions (including joint sense, position sense, and vibration sense) were intact. Patellar reflexes were brisk, and the Achilles tendon reflex was bilaterally extinguished. Sphincter control was normal. The modified Barthel index upon admission was 55/100 signifying severe dependence.

The rehabilitation regimen involved balneotherapy in the mineral bath at a temperature of 35° for 30 minutes, exercises in water, individual kinesitherapy, electrotherapy with galvanic and exponential current, and paraffin therapy. Our thermal water is homeothermal, slightly radioactive, with a temperature range of 34-38°C, and with a high concentration of sodium, calcium, and magnesium cations, as well as potassium in smaller quantities. Among the anions, sulfates, hydrogen carbonates, and chlorine are the most significant. The presence of fluorine is also noteworthy. Microelements are represented in a vibrant spectrum. The presence of lithium, cobalt, and copper, as well as zinc, strontium, barium, manganese, molybdenum, cesium, and selenium, has been determined. The kinesiotherapy program consisted of actively supported and active exercises to maintain the range of motion. Resistance exercises were gradually introduced for muscles with a score of 3/5 on the manual muscle test. Before the exercises, we utilized paraffin wraps as a preparation and warm-up for kinesiotherapy. Galvanization was applied longitudinally for 10 days, followed by a pause of the same duration. We used electrostimulation with triangular impulses (E1 form: exponential pulse duration of 250 ms, pause duration of 500 ms) for the feet' dorsiflexors, utilizing a bipolar technique. After 10 sessions, we took a 10-day break.

After a total of 35 days of rehabilitation, the patient was able to ambulate with the walker independently and ascend and descend stairs with minimal supervision. Gross muscle strength had improved by one grade in all muscle groups of the lower extremities, as shown in Table 1. The modified Barthel index upon discharge was 81/100 indicating moderate dependence.

**Table 1:** Gross motor strength and modified Barthel index on admission and discharge.

Gross motor strength	Upon admission	Upon discharge
Hip flexors and extensors	3/5	4/5
Knee flexors and extensors	3/5	4/5
Dorsal and plantar flexors	2/5	3/5
Modified Barthel index	55/100	81/100

Case two

A 47-year-old male, with no prior medical history, developed mild symptoms of infection and tested positive for COVID-19. Fifteen days after the infection, he began experiencing tingling and numbness in both feet. Symptoms gradually progressed to his lower and upper legs, followed by progressive weakness over the next two months first in distal and then in proximal muscle groups of lower extremities. After two months, he lost balance and the ability to walk. The patient was initially admitted to the neurological department of a local hospital, where an MRI of his brain and cervical spine showed no abnormalities. Treatment with pulse corticosteroid therapy for five days had no effect, and he was referred to a neurological clinic for further diagnostic testing. The patient was diagnosed with chronic inflammatory demyelinating polyradiculoneuropathy and treated with plasmapheresis.

Upon admission to our rehabilitation center, he presented with a paraparetic gait pattern and required two forearm crutches for short distances. Gross motor power in the upper extremities was preserved. Reflexes of the upper extremities were within normal limits. Gross motor strength scores on manual muscle testing were as follows: hip flexors and extensors 3/5, knee flexors and extensors 3/5, plantar flexors of feet 2/5, and dorsal flexors 1/5. Paresthesias below the Th9 level were present with hyperreflexia in the lower extremities. Sphincter control was preserved. The rehabilitation protocol was balneotherapy in a mineral bath of temperature 35° (half hour), combined with exercises in water, individual kinesitherapy, and electrotherapy with galvanic currents. The individual kinesiotherapy regimen included passive exercises aimed at preserving the range of motion in both ankle joints, complemented by actively supported and active exercises targeting the maintenance of mobility in the knees and hips bilaterally. Progressive resistance exercises for muscles that had scored 3/5 on manual muscle tests were systematically introduced. Daily balance training exercises were implemented. Additionally, the patient underwent gait training with a mobility aid. We used descending galvanization in cycles of 10 days, with a break of 10 days in between.

After undergoing 36 days of rehabilitation, there has been a significant improvement in gross motor power by half to one grade, depending on the muscle group, as shown in Table 2. Upon admission, the modified Barthel index was 60/100, indicating severe dependence, and upon discharge 78/100, indicating moderate dependence.

**Table 2:** Gross motor strength and modified Barthel index on admission and discharge.

Gross motor strength	Upon admission	Upon discharge
Hip flexors and extensors	3/5	4/5
Knee flexors and extensors	3/5	4-/5
Dorsal flexors	1/5	2/5
Plantar flexors	3/5	3+/5
Modified Barthel index	60/100	78/100

Case three

A 49-year-old male, with a history of high blood pressure, experienced sudden urinary retention, tingling, and weakness in his legs. Ten days prior, he had a mild fever, but did not go to the doctor as he was feeling relatively well. Due to suspicion of Guilian-Barré syndrome, he was hospitalized in the infectious department of a local hospital. A PCR test for COVID-19 came back positive. Because of the progression of neurological symptoms (paraplegia with urinary retention), he was referred to the neurological clinic for further diagnostic treatment and diagnosed with acute transverse myelitis. The patient was treated with pulse corticosteroid therapy for five days, followed by plasmapheresis for five cycles.

He underwent the first course of rehabilitation, which lasted 21 days. Upon admission, the patient was immobile with the inability to maintain balance while sitting. Upper gross motor strength was 3/5 on the manual muscle test. Hypotrophy of the thenar and hypothenar muscles led to incomplete extension of the fingers. On the lower extremities, gross motor strength was 0/5 with hypertonia and hyperreflexia. Urinary retention was present. The patient required suppositories to induce bowel movements. He expressed feelings of sadness, nervousness, and a lack of motivation. The rehabilitation protocol entailed a course of balneotherapy in a mineral bath set at a temperature of 34°C, individual kinesitherapy, galvanization, hand massage, and paraffin therapy. Kinesitherapy was focused on passive exercises to maintain the range of motion of the lower extremities, as well as active exercises with resistance for the upper extremities. To increase functionality, emphasis was placed on exercises performed in bed, including rolling from side to side and seated balance exercises. The goal for the patient was to master wheelchair-to-bed transfers and vice versa.

After discharge, his upper extremity manual muscle strength was 4/5 on the manual muscle test. His balance while sitting was better, but he still needed the maximum help of two people in transfers from bed to wheelchair. Barthel index at admission was 10/100 (total dependence) and at discharge 15/100 (total dependence).

The second rehabilitation session started two months after the initial program and lasted for 15 days. At admission, the patient was able to sit and maintain balance for a short duration but needed help from two people in transfers from the bed to the wheelchair. On the manual muscle test, gross motor strength was 4/5 in the upper extremities, 2/5 in the knee extensors of the right leg, and 0/5 in all other muscle groups, as shown in Table 3. Grade 3/4 hypertonia by the modified Aschowt scale in the left leg and grade 2/4 in the right leg were noted with clonus present in both. The patient had a good mood and increased motivation during this rehabilitation phase and was educated on self-catheterization. The rehabilitation protocol was similar to the previous program. At discharge, the patient needed minimal assistance from one person to move from bed to wheelchair and was able to turn to the side independently. The modified Barthel index score at discharge was 27/100, indicating severe dependence.

**Table 3:** Gross motor strength and modified Barthel index before and after rehabilitation.

Gross motor strength	Upon admission	After two courses of rehabilitation
Upper extremities	3/5	4/5
Hip flexors and extensors	0/5	0/5
Knee flexors	0/5	0/5
Knee extensors-right leg	0/5	2/5
Knee extensors-left leg	0/5	0/5
Dorsal flexors	0/5	0/5
Plantar flexors	0/5	0/5
Modified Barthel index	10/100	25/100

## Discussion

GBS is an autoimmune neuropathy manifested by progressive and symmetrical muscle weakness or sensory loss. Molecular mimicry, antiganglioside antibodies, and likely complement activation are involved in GBS pathogenesis [6]. GBS was frequently a post-infectious illness associated with *Campylobacter jejuni, Mycoplasma pneumoniae, *and *Haemophilus influenzae,* as well as Epstein-Barr virus, influenza A, cytomegalovirus, hepatitis E, enteroviruses, and Zika virus [7]. As for SARS-CoV-2-associated GBS, there is a theory that COVID-19 stimulates inflammatory cells and the production of various inflammatory cytokines, resulting in the initiation of immune-mediated processes [8]. As reported in the literature, GBS symptoms began on average 19 days after the onset of COVID-19 infection [9]. In the case of our female patient, the first symptoms started 15 days after infection. Radišić et al. [10] showed that there was no difference in clinical and electrophysiological features, disease course, and outcome between post-COVID-19 and non-COVID-19 GBS patients. As for rehabilitation outcomes of post-COVID-19 GBS patients, there are limited data. In the study of Marcello Solaro et al. [11], COVID-19-related GBS had a better clinical outcome than non-COVID-19-GBS. However, epidemiological considerations cannot be deemed due to the small sample. Similar results were reported by Massucio et al. [12] in a study that included 11 patients. Although the current literature says that AMAN forms of GBS have less favorable outcomes in comparison to the demyelinating form in the case of our female patient, she had a favorable outcome after rehabilitation and was moderately dependent on activities of daily living.

Our second case of chronic inflammatory demyelinating polyradiculoneuropathy represents a rare neurological complication of COVID-19 disease. The main difference between chronic inflammatory demyelinating polyradiculoneuropathy (CIDP) and acute inflammatory demyelinating polyradiculoneuropathy (AIDP) is the time course. CIDP has a slower progression and all published CIDP criteria require longer than eight weeks to differentiate CIDP from AIDP based on time to greatest weakness [13]. Reports about COVID-19 CIDP are rare. Patel et al. [14] reported a case of a 69-year-old female with post-COVID chronic demyelinating polyradiculoneuropathy. We could not find data about rehabilitation outcomes. In our case, rehabilitation was successful as the patient achieved moderate independence in activities of daily living.

Limited data are available on the incidence of acute transverse myelitis resulting from COVID-19. The current evidence consists of a small number of case reports. Some proposed pathways by which SARS-CoV-2 could affect the spinal cord are direct invasion of the spinal cord, cytokine storm, or an autoimmune response [15]. A possible mechanism for post-infectious acute transversal myelitis is similar to GBS, molecular mimicry, where immune-mediated injury to the nervous system occurs due to the production of autoantibodies [16]. Acute therapeutic options for acute transverse myelitis include corticosteroids, plasma exchange, IV immunoglobulin, and chemotherapeutic agents such as cyclophosphamide. In some instances, combinations of these therapies are used [17]. In non-COVID transverse myelitis cases, the results of long-term follow-up studies indicate that nearly one-third of patients recover with little to no impairment. Another one-third show a moderate level of disability, which may include independent ambulation with mild spasticity, manageable urinary/bowel changes, and some sensory deficits. The remaining one-third of patients experience severe disability [18]. In the study of Ali et al. [19], which included 131 patients, 63.4% of cases had poor recovery with significant neurological deficits, and 9.6% of patients with transverse myelitis were completely paralyzed. Rehabilitation has to be introduced very early in the course of illness and should continue for the long term, as the recovery process for acute transverse myelitis can go on for a year or more [18,20]. Our patient had an improvement after 36 days of rehabilitation, but he was still severely dependent on activities of daily living. It is still early to conclude as he was recommended for additional physical therapy during the first year of recovery. It is noteworthy that all three patients experienced a mild infection, followed by serious neurological complications 10-15 days later.

## Conclusions

COVID-19 is known to induce a wide spectrum of neurological symptoms, with varying degrees of severity. To provide optimal care for COVID-19 patients with neurological sequelae, a multidisciplinary approach is essential. Rehabilitation has been demonstrated to offer significant benefits, including increased independence and improved quality of life. Our patients made functional improvements between admission and discharge after five weeks of a rehabilitation program.

The health resort setting appears to be suitable and safe for the rehabilitation of patients with neurological complications of COVID-19. However, further research with a larger cohort is necessary to draw a conclusion.
